# A new species of *Dystacta* Saussure, 1871 from Nyungwe National Park, Rwanda (Insecta, Mantodea, Dystactinae)

**DOI:** 10.3897/zookeys.410.7053

**Published:** 2014-05-20

**Authors:** Riley Tedrow, Kabanguka Nathan, Nasasira Richard, Gavin J Svenson

**Affiliations:** 1 Department of Invertebrate Zoology, Cleveland Museum of Natural History, 1 Wade Oval Drive, Cleveland, Ohio, USA; 2 Department of Biology, Case Western Reserve University, 10900 Euclid Avenue, Cleveland, Ohio, USA; 3 Kitabi College of Conservation and Environmental Management (KCCEM), C/O Rwanda Development Board, P.O Box 330 Huye, Kigali, Rwanda

**Keywords:** Mantodea, praying mantis, *Dystacta*, Afrotropical, taxonomy, new species

## Abstract

A recent targeted entomological survey in the Republic of Rwanda has produced two conspecific male and female specimens of an undescribed species of praying mantis (Mantodea). The specimens were collected in Nyungwe National Park in May of 2013. The species is closest morphologically to *Dystacta alticeps* (Schaum, 1853). Therefore, a new species is described, *Dystacta tigrifrutex* **sp. n.**, along with the first instar nymphs and ootheca. In addition, the previously monotypic genus *Dystacta* Saussure, 1871 is re-described to provide a broader definition of the genus group. Habitus images, measurement data, a key to species, natural history information, and locality data are provided.

## Introduction

The order Mantodea is a diverse group of predatory insects known as praying mantises. The group is comprised of approximately 2,500 described species (Ehrmann 2002) with a vast array of morphological and behavioral adaptations. Although the order has received much more taxonomic attention in recent years (see Rivera 2010), it has long been a neglected order of insects with strong potential for new species discovery (e.g. Svenson 2014).

**Figure 1.**
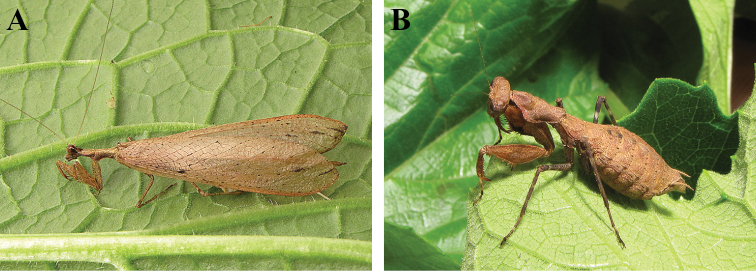
*Dystacta tigrifrutex* sp. n., live habitus: **A** holotype male (GSMC004381) **B** allotype female (GSMC004420).

During a preliminary survey of insects in Nyungwe National Park (NNP) in southwestern Rwanda, a single male and single female of an unknown species of praying mantis were collected (Fig. 1). The male was attracted to a metal halide light system and was found on the ground near the trap. The female was found in close proximity to the light walking through ground vegetation. It was not clear if the female was attracted to the light, but the male is assumed to have flown in specifically to the light. The collecting site was in a high altitude location within NNP (~2,500 m) in montane forest. The weather was cool (~15 °C) with intermittent rain.

After a thorough examination of the new specimens, we determined them to be representative of an undescribed species belonging to the monotypic genus *Dystacta* Saussure, 1871. Although distinct from the only other species in the genus, *Dystacta alticeps* (Schaum, 1853), our new species shares a number of similarities justifying its inclusion within the genus. Originally described by Saussure to contain a single species, *Dystacta paradoxa* Saussure, 1871, that was later synonymized with *Mantis alticeps* Schaum, 1853 (now within *Dystacta*), four additional species have been described within *Dystacta*, three being later synonymized with *Dystacta alticeps* and the other moved to a different genus (see below). Considering the broad range of *Dystacta alticeps* with records in Angola, Botswana, Congo, Malawi, Mozambique, Namibia, South Africa, Tanzania, and Zimbabwe (Ehrmann 2002), it is not surprising that synonymies have been described. *Dystacta* is classified within the tribe Dystactini with seven other genera (Ehrmann 2002). Svenson and Whiting (2009) and Yager and Svenson (2008) recovered the tribe within a diverse assemblage including species of Amelinae, Chroicopterinae, and Hapalomantinae, a result strongly supported by molecular data.

We now describe the new species using male and female specimens as well as characterize the ootheca and first instar nymphs. In addition, because *Dystacta* was originally described based on a single species and the description was too narrow and reflected only the variability within *Dystacta alticeps*, we redescribe the genus herein to provide a more accurate set of characters for the genus-group.

## Methods

### Region sampled

Nyungwe National Park is Africa’s largest protected mountain rainforest, covering about 970 km² of pristine habitat. The forest is located in the Albertine Rift, a series of mountain ranges stretching from the Rwenzori mountains in western Uganda to the Lendu Plateau in eastern Congo. The park resides in the southwestern portion of Rwanda. The rainy season for Nyungwe lasts from September to May, and the dry season from June to August (Kaplin 2013). Nyungwe has an average minimum temperature of 10.9 degrees Celsius and and average maximum temperature of 19.6 degrees Celsius. The forest is complex and diverse, supporting a variety of soil types that allow for tall forests and dry ridges with short trees and thickets. Bamboo covers most of the southeastern part of the park, but open herbaceous areas, flooded forests, and marshes are also scattered throughout (Plumptre et al. 2002). The forest elevation ranges from 1,600–2,950 meters and it recieves rain in excess of 2000 mm per year (http://nyungwepark.com/nyungwe/the-park/).

### Collection and preparation

A metal halide light trapping system was utilized at night to attract insects. The system is composed of tent poles holding up a white curtain in a vertical position. A copper “T” sits perpendicularly atop the center of the apparatus. A metal halide bulb is affixed to each end of the horizontal line of the “T”. These bulbs are supported by individual light ballasts and powered by either an outlet or a small portable generator. Insects are attracted to the light and land upon the white curtain. Specimens were placed in vials directly from the curtain. These lights typically attract flying and nonflying insects to the perimeter of the light apparatus, and a scan often produces many more specimens collected via sweep net or hand.

The male and female were kept alive in cubical screen enclosures to monitor their behavior and capture live habitus images. The male died within 12 hours of captivity. The female produced a single ootheca that was incubated in a humid container in order to procure first instar nymphs. The female lived in captivity for two weeks then had to be euthanized for transport back to the United States. The female was preserved in ethanol and later pinned due to her large abdomen that may have spoiled in the field or during transport. The male was field pinned after a leg was preserved in ethanol for future genetic work (tissue sample deposited in the Cleveland Museum of Natural History).

### Descriptive conventions and character systems

The species treatment within this study provides a brief diagnosis, new natural history observations, and verbal character descriptions stemming from the anterior surface of the head, the dorsal surface of the pronotum, the prothoracic leg, the wings, and the abdomen. The verbal descriptions are provided for the male and female as well as the ootheca and the first instar nymphs. Description of ootheca is modeled after Breland and Dobson (1947). Foreleg spine nomenclature follows Wieland (2008, 2013) where diagrams of arrangements can be viewed.

*Measurements* Specimens were measured using a Leica M165C stereo-microscope and an IC80 HD coaxial video camera using the live measurements module of the Leica Application Suite (LAS). All measurements presented in this study are in millimeters. A total of 21 measurement classes were captured including:

1. *Body length* = length of body from central ocelli to posterior tip of wing or abdomen (intraspecifically variable measurement, primarily for general size estimation).
2. *Forewing length* = from proximal margin of axillary sclerites to distal tip of the discoidal region.
3. *Hindwing length* = from proximal margin of axillary sclerites to distal tip of the discoidal region.
4. *Pronotum length* = from anterior margin to posterior margin.
5. *Prozone length* = anterior margin of pronotum to center of supra-coxal sulcus.
6. *Pronotum width* = from lateral margins at the widest point, the supra-coxal bulge.
7. *Pronotum narrow width* = from lateral margins of the pronotum at narrowest region of metazone.
8. *Head width* = from lateral margins of the eyes at widest point.
9. *Head vertex to clypeus* = from the vertex of the head at center to the lower margin of the frons and upper margin of clypeus.
10. *Frons width* = from lateral margins of frons, inferior to the antennal insertions, at the widest point.
11. *Frons height* = from upper margin abutting central ocellus to lower margin abutting clypeus.
12. *Prothoracic femur length* = from proximal margin abutting trochanter to distal margin of genicular lobe.
13. *Mesothoracic femur length* = from most proximal margin abutting trochanter to the distal side of the terminal spine insertion site.
14. *Mesothoracic tibia length* = from most proximal groove near joint with the femur to the distal side of the terminal spine insertion site.
15. *Mesothoracic tarsus length* = from proximal joint to the apex of the ungues curve.
16. *Metathoracic femur length* = from most proximal margin abutting trochanter to the distal side of the terminal spine insertion site.
17. *Metathoracic tibia length* = from most proximal groove near joint with the femur to the distal side of the terminal spine insertion site.
18. *Metathoracic tarsus length* = from proximal joint to the apex of the ungues curve.
19. *Anteroventral femoral spine count* = all inner marginal ridge spines and two proximal near marginal spines, but excluding the genicular spine.
20. *Anteroventral tibial spine count* = all inner marginal ridge spines, but excluding the distal terminal spur.
21. *Posteroventral tibial spine count* = all outer marginal ridge spines, but excluding the distal terminal spur.

The measurement of total body length was taken from the central ocellus to tip of posterior margin of abdomen or wing, which produced a measure only useful for general assessment of body size rather than species description. Since head position, abdominal expansion, and wing position are all variable, total body length should only be used as a rough measure class to initially discriminate between the small and large praying mantis species when performing identifications.

*Imaging* Live habitus images were captured with a Canon Powershot SX10 IS with accompanying Canon Speedlite 430EX II flash units. High resolution images of the types, the pronotum, and head images were captured using a Passport Storm© system (Visionary Digital™, 2012), which includes a Stackshot z-stepper, a Canon 5D SLR, macro lenses (50mm, 100mm, and MP-E 65mm), three Speedlight 580EX II flash units, and an associated computer running Canon utility and Adobe Lightroom 3.6 software. The z-stepper was controlled through Zerene Stacker 1.04 and images were processed using the P-Max protocol. All images were captured over an 18% grey card background for white balance standards. Images were processed in Adobe Photoshop CS6 Extended to adjust levels, contrast, exposure, sharpness, and add scale bars. Minor adjustments were made using the stamp tool to correct background aberrations and to remove distracting debris. Plates were constructed using Adobe Illustrator CS6. Habitus images of the types can be viewed online at http://specimens.mantodearesearch.com.

## Taxonomic placement

Afrotropical distributed species classified within Amelinae, Chroicopterinae, and Dystactinae (*sensu* Ehrmann 2002) were compared with the male and female of the undescribed species (*Dystacta tigrifrutex* sp. n.) discovered in NNP. In addition, available keys by Giglio-Tos (1927) and Kaltenbach (1998) were used to recover genus level identification. Both methods proved that the new taxon had the greatest affinities with *Dystacta alticeps* (Schaum, 1853), and should be included within the monotypic genus *Dystacta* Saussure, 1871. The holotype female of *Dystacta alticeps* was examined and compared with the female specimen from NNP and though they are remarkably similar, they are unique (for reference, the imaged type of the junior synonym, *Paracilnia ornatipennis* Beier, 1935, can be seen at http://specimens.mantodearesearch.com). In addition, a close comparison with holotype of *Pseudodystacta braueri* Karney, 1908, another very similar species that was previously included within *Dystacta* (see below), revealed key differences. For example, *Dystacta tigrifrutex* has a much more elongate pronotum and a greater constriction in the metazone following the supra-coxal dilation than seen in *Pseudodystacta braueri*; the male wings are densely ciliated along the anterior margin and in the costal region while not densely ciliated in *Pseudodystacta braueri*; the medial and cubital veins of the male forewing are divergent and widely spread in *Dystacta tigrifrutex* while closer together and near parallel in *Pseudodystacta braueri*: the female is apterous while *Pseudodystacta braueri* females have very short and rounded wings with blunt ends; and finally, the females have two large tubercles near the posterior margin of the pronotum that are not present in *Pseudodystacta braueri*.

Many characters easily define *Dystacta alticeps* and *Dystacta tigrifrutex* as congeneric including: dense ciliation along the anterior margin and in the costal region of the forewings; the medial and cubital veins of the forewings are divergent and widely spread; a vertex that is slightly curved in males and strongly curved in females; the male antennae ciliated and medium to long; the female antennae short; the wings of the male partially opaque, extending well beyond the abdomen; the wings of females not fully developed; the forefemora with 12–13 anteroventral femoral spines; the foretibiae with 10-11 anteroventral tibial spines; and the supra-anal plate transverse. However, since *Dystacta* is monotypic, we have determined that its current description primarily reflects *Dystacta alticeps* and required a more broad description to include the new species.

### Dystacta

Saussure, 1871

http://species-id.net/wiki/Dystacta

Dystacta : Saussure 1871: 455; Stål 1877: 51; Westwood 1889: 17; Kirby 1904: 226; Giglio-Tos 1927: 206; Beier 1935: 21; Beier 1964: 947; Beier 1968: 10; Ehrmann 2002: 124; Otte and Spearman 2005: 30.

#### Genus-type.

*Dystacta paradoxa* Saussure, 1871 (by monotypy). The genus-type is currently the junior synonym of *Mantis alticeps* Schaum, 1853.

#### Taxonomic history.

Henri de Saussure created the genus *Dystacta* in 1871 (pg. 445) for a male specimen collected by M. Brunner de Wattenwyl in South Africa, *Dystacta paradoxa* Saussure, 1871. Later, Westwood (1889: 17) included *Mantis alticeps* Schaum, 1853 (pg. 113) within *Dystacta* (it should be noted that the date of Schaum’s description of *Mantis alticeps* is incorrect in Otte and Spearman (2005) as they list it as 1852). Kirby (1904: 226) later synonymized *Dystacta paradoxa* Saussure, 1871, with *Mantis alticeps* Schaum, 1853. Four more species were subsequently described, three were eventually synonymized with *Mantis alticeps* by Beier (1935: *Polyspilota marmorata* Schulthess, 1899 and *Dystacta stali* Karny, 1908) and Kaltenbach (1996: *Paracilnia ornatipennis* Beier, 1935). The fourth, *Dystacta braueri* Karney, 1908, was removed from *Dystacta* and fixed as the type species of a new genus, *Pseudodystacta*, erected by Kaltenbach (1996). Therefore, *Dystacta* has always effectively been monotypic until now. The genus and included species, particularly *Dystacta alticeps*, have been included in various taxonomic works and checklists throughout the 1900’s.

#### Redescription.

**Male.** *Body*: Ochre to dark brown with black markings; head lacking projections, the antennae simple; the pronotum almost spade-like with a strong supra-coxal dilation; the wings smoky grey or brown, extending beyond the abdomen; the meso- and metathoracic legs of a similar length; the abdomen tubular.

*Head*: Transverse, eyes slightly exophthalmic; the vertex rounded or slightly rounded, the parietal sutures present. Juxta-ocular protuberances absent. Ocelli large, protruding from small cuticular mounds, but the region between all three slightly raised with a triangular shape; the lateral ocelli oriented outward; the region around the raised ocelli and below the frontal suture depressed. The clypeus transverse, the upper margin convex; the lower margin slightly concave. Labrum rounded. The flagellomeres of the antennae mostly pale in the basal half, transitioning to a darker coloration on the distal end of the antennae. Anterior surface of head mostly pale or ochre with dark speckling across the surface; frons ochre with dark splotches of black, a transverse black band just below the antennae extends laterally across the anterior surface of the eyes; the clypeus ochre with dark markings; labrum ochre, occasionally with darker markings; mandibles ochre; vertex ochre with dark splotches or banding; maxillary palpi ochre with a darkened terminal segment.

**Figure 2.**
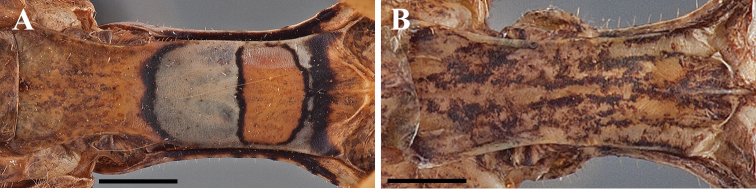
*Dystacta* male prosternum (scale bar = 1 mm), posterior margin on the left: **A** *Dystacta alticeps* Schaum, 1853 **B** *Dystacta tigrifrutex* sp. n.

*Thorax*: Longer than wide, with an expanded supra-coxal bulge; dorsal surface smooth. Medial region of prozone peaked, sloping to the anterior margins; medium length with margins gradually tapering anteriorly to a rounded anterior margin; the margins smooth, but with setae present. Metazone with two tubercles positioned on each side of the medial line just anterior to the posterior margin, a raised carina oriented anterolaterally, extending to the lateral margins of the metazone. Metazone with concave lateral margins tapering posteriorly until two thirds from the supra-coxal bulge, then widening to the posterior margin; margins smooth, but with setae present; the dorsal surface of the metazone not depressed. Ochre with faint black markings. Prosternum with or without a complex black and whitish pattern (Fig. 2); if present, a transverse black band anteriorly and a curved black band posteriorly, the posterior half of the medial region whitish colored, the anterior half with a trapezoidal shape of brown surrounded by a thin black band, the trapezoid reaching the lateral margins. The wings elongate, extending well beyond the terminus of the abdomen. Forewings opaque; setae along the anterior margin, the costal region densely ciliated, the discoidal region ciliated or not; the discoidal region smoky brown or grey with dark splotches; the veins more pigmented than surrounding cell colors; the medial and cubital veins are divergent and widely spread. Hindwings with setae along anterior margin; mostly matching coloration of forewing; the wings extending beyond the abdomen.

*Prothoracic Legs*: Femur shape normal with a straight dorsal margin; spines robust, pale proximally and black distally; femoral groove to accommodate the tibial spur in the proximal half; the posterior surface smooth; 4 discoidal spines. Posterior surface of femur ochre with black stippling; setae dispersed across a pale ventral surface. The discoidal spines robust, the third from the base very large and robust, twice the length of the second and fourth. Anteroventral femoral spines alternating between short and long, the longer spines of similar length and the shorter spines of similar length; posteroventral femoral spines all of the same length; the posterior and anterior genicular spines small, but robust. Tibia with sparse or dense setae along the dorsal margin and on the posterior, anterior and ventral surfaces; anterior and posterior surfaces ochre with darker markings. Posteroventral tibial spines of similar length, except for the most distal spine being larger than the others; anteroventral tibial spines gradually increase in length from the most proximal to the most distal spine. Forecoxae mostly smooth with setae interspersed throughout, a few tubercles present along the margins, but none are robust as seen in females.

*Meso- and Metathoracic Legs*: Femora with ventral (posterior) carina well developed; dorsal (anterior) carina absent; surface with numerous small, fine setae; darkly speckled with black markings. Coxae with numerous black markings speckling the surface. Tibia round, covered with setae. Tarsi with ample setae.

*Abdomen*: Smooth, tubular with brownish to black coloration; the surface with numerous setae across the surface. Tergites rounded at the postero-lateral margins. Supra-anal plate transverse, with a rounded terminus. Cerci with ample setae, round, tapering to a point.

**Female.** *Body*: Medium; ochre to dark brown with black markings; a highly convex head; the pronotum almost spade-like with strong supra-coxal dilation; the wings reduced or absent; the meso- and metathoracic legs of a similar length; the abdomen broad and elliptical.

*Head*: Transverse, eyes not particularly exophthalmic; vertex highly convex, the parietal sutures present; juxta-ocular protuberances absent. Frontal suture faint, but forming dorsally oriented curve. Ocelli absent or vestigial, but lenses not visible; a carina connecting former ocellar locations that connects all three and forms a U; region within the U and below the frontal suture depressed. Frons transverse with an angled carina below the antennae, meeting medially with a raised point. Clypeus transverse, the upper margin convex, the lateral margins tapering to a rounded distal terminus; surface with a medial, transverse carina. Labrum rounded with a darkened distal terminus. Antennal flagellomeres pale basally and fading to black toward the distal end; setae present. Anterior surface of head mostly pale or ochre with tiny dark speckling across the surface; labial palpi ochre, maxillary palpi ochre, sometimes with black markings.

*Thorax*: Longer than wide, with an expanded supra-coxal bulge; dorsal surface with uneven tiny depressions and scattered black tubercles. Prozone with medial region of prozone peaked, sloping to the lateral margins. Metazone with uneven sculpting across the surface. Margins of the prozone convex, tapering sharply to a narrowed, rounded anterior margin; margins of metazone strongly convex, tapering to a narrow constriction medially before widening to the posterior margin. Tubercles present on the margins of the prozone; a few large tubercles present on the margins of the metazone. The supra-coxal sulcus strongly defined and sweeping anteriorly prior to the expanded lateral margins at the supra-coxal bulge. Prosternum with or without a complex black and whitish pattern (Fig. 2); if present, a transverse black band anteriorly and a curved black band posteriorly, the posterior half of the medial region whitish colored, the anterior half with a trapezoidal shape of brown surrounded by a thin black band, the trapezoid reaching the lateral margins. Wings reduced or absent.

*Prothoracic Legs*: The femur squat, almost forming a triangle; spines robust, pale proximally and black distally; femoral groove to accommodate the tibial spur proximal to the middle. The posterior surface of the femur with a marginal carina; dorsal margin narrowing; the posterior surface with numerous small tubercles; 4 discoidal spines. The ventral surface of the femur pale with numerous tubercles medially distal to the discoidal spines, the tubercles continue just proximal to the discoidal spines until the junction with the trochanter; the discoidal spines robust, the third from the base very large and robust. The anterior surface of the femur mostly pale with some black marking. Tibia robust with rare, fine setae on the surface and near the lateral margins of the ventral surface; posterior surface pale with some black markings; ventral surface ochre and lustrous; posteroventral and anteroventral tibial spines gradually becoming longer from the proximal to the distal end. Coxae with tubercles and setae across the surface, the dorsal margin with setae and a few strong tubercles; the anterior surface mostly ochre with some black markings, the distal lobes ochre.

*Meso- and Metathoracic Legs*: Femora with ventral (posterior) carina well developed; dorsal (anterior) carina absent; surface with numerous small, fine setae. Coxae with numerous black markings speckling the surface. Tibia round, covered with setae. Tarsi short with ample setae.

*Abdomen*: Very broad, elliptical, the widest being the middle. Fine setae disperse across the dorsal and ventral surfaces; each tergite with a medial keel, more pronounced anteriorly. Tergites rounded at the posterolateral margins. Supra-anal plate transverse, with a rounded terminus. The ovipositor enlarged and broad, projecting far beyond the distal margin of the supra-anal plate and the cerci. Cerci round, tapering to a point.

#### Key to species

**Table:** 

1	Male and female large in size (observed range 44–56 mm). Forewings of male with setae, some robust, along anterior margin; the surface of the costal region ciliated; the discoidal region smooth, not ciliated. Male and female with distinct pattern on the prosternum (Fig. 2A). Female brachypterous. Male foretibiae with 9–10 posteroventral spines. Male forefemora with 13 anteroventral spines	*Dystacta alticeps* (Schaum, 1853)
1’	Male and female small in size (observed range 19–34 mm). Forewings of male with dense setae along anterior margin; the surface of the costal and discoidal regions tightly ciliated. Male and female prosternum ochre with dark brown speckling that becomes more dense posteriorly (Fig. 2B). Female apterous. Male foretibiae with 7–8 posteroventral spines. Male forefemora with 12 anteroventral spines	*Dystacta tigrifrutex* sp. n.

### Dystacta alticeps

(Schaum, 1853)

Mantis alticeps : Schaum 1853: 777; Schaum 1862: 113.
Cardioptera alticeps : Saussure 1871: 211.
Dystacta alticeps : Westwood 1889: 17; Krauss 1901: 284; Kirby 1904: 226; Karny 1908: 371; Rehn 1912: 112; Werner 1923: 118; Giglio-Tos 1927: 207; Beier 1935: 21; Kaltenbach 1996: 239; Lombardo 1997: 73; Kaltenbach 1998: 19; Ehrmann 2002: 124; Otte and Spearman 2005: 31.
Dystacta paradoxa : Saussure 1871: 447; Saussure 1872: 80; Stål 1877: 51; Westwood 1889: 17; Kirby 1899: 347 [SYN of *Dystacta alticeps*]; Ehrmann 2002: 124 [SYN]; Otte and Spearman 2005: 31 [SYN].
Polyspilota marmorata : Schulthess 1899: 192; Kirby 1904: 241; Beier 1935: 21 [SYN of *Mantis alticeps*]; Ehrmann 2002: 124 [SYN; Otte and Spearman 2005: 31 [SYN].
Paracilnia ornatipennis : Beier 1935: 102; Kaltenbach 1996: 239 [SYN of *Mantis alticeps*]; Ehrmann 2002: 124 [SYN]; Otte and Spearman 2005: 31 [SYN].
Dystacta stali : Karny 1908: 371; Beier 1935: 21 [SYN of *Mantis alticeps*]; Ehrmann 2002: 124 [SYN]; Otte and Spearman 2005: 31 [SYN].

#### Repository.

Holotype Female. Museum für Naturkunde der Humboldt-Universität, Berlin, Germany.

### Dystacta tigrifrutex

Tedrow & Svenson sp. n.

http://zoobank.org/BA908752-636F-4B12-9510-A54E75E9B2B9

http://species-id.net/wiki/Dystacta_tigrifrutex

#### Repository.

Holotype Male (CLEV GSMC004381). Allotype Female (CLEV GSMC004420). The Cleveland Museum of Natural History, Cleveland, OH, USA

Holotype and Allotype labels: Pinned. Rwanda, Nyungwe National Park, -2.478121, 29.200055, 2446 m, 4–9 May 2013, Coll: R. Tedrow & G.J. Svenson.

#### Natural history.

Based on the collecting location on the ground for both the male and the female and the fact that the female is apterous, we presume that the species utilizes a low vegetation or forest floor habitat. This would be consistent with morphologically similar species in Africa (Chroicopterinae), Asia (some Amelinae) and South America (some Thespinae). The female walks nimbly through vegetation and uses her forelegs periodically, pausing and moving them similar to boxing mantises (species of *Hestiasula*). The male took flight readily and was difficult to observe and expired quickly in captivity.

#### Diagnosis.

Male brown with elongate wings. The easiest way to distinguish *Dystacta tigrifrutex* from *Dystacta alticeps* is the distinct black and whitish pattern on the prosternum of *Dystacta alticeps* (Fig. 2A) while *Dystacta tigrifrutex* has a speckled pattern on the prosternum (Fig. 2B). In addition, the presence of tight ciliation on the discoidal region of the forewings of male *Dystacta tigrifrutex* compared with the smooth surface seen in *Dystacta alticeps*. Females are apterous in *Dystacta tigrifrutex* and brachypterous in *Dystacta alticeps*.

#### Description.

**Figure 3.**
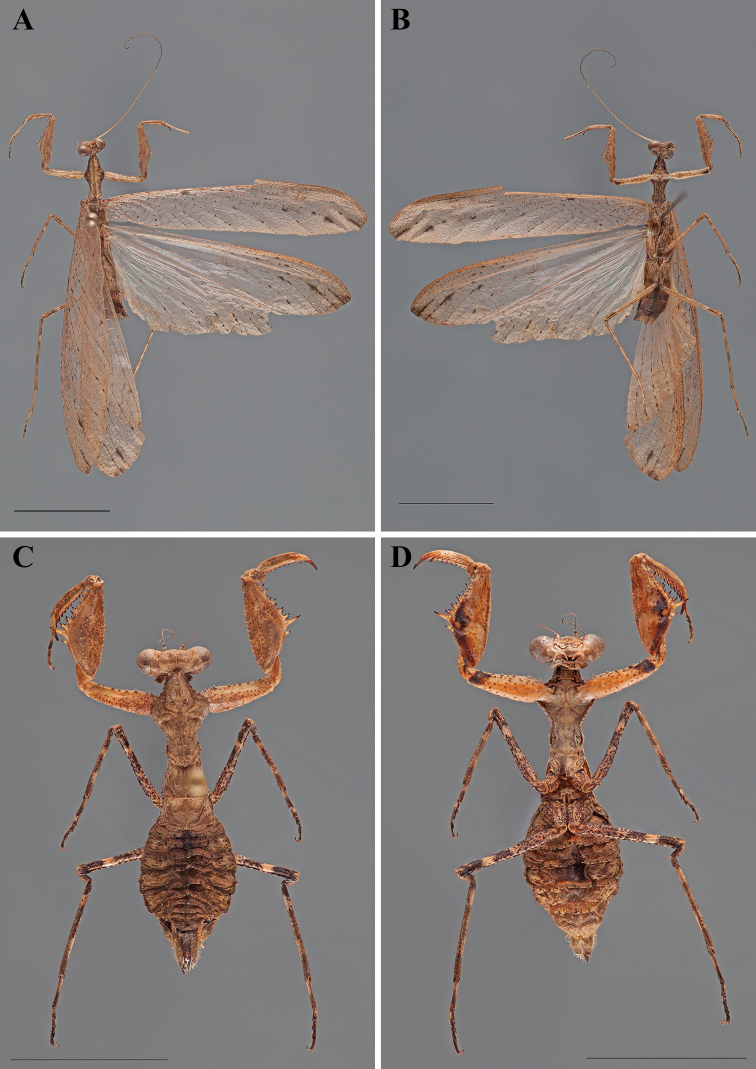
*Dystacta tigrifrutex* sp. n., pinned habitus (scale bar = 1 cm). Holotype male (GSMC004381): **A** dorsal **B** ventral. Allotype female (GSMC004420): **C** dorsal **D** ventral.

**Male.** **Holotype** (Figs 1A, 3A–B). Body length 34.72; forewing length 28.2; hindwing length 27.16; pronotum length 4.65; prozone length 1.77; pronotum width 2.11; pronotum narrow width 1.03; head width 3.46; head vertex to clypeus 1.53; frons width 1.27; frons height 0.595; prothD. tigrifrutex D. tigrifrutex oracic femur length 5.19; mesothoracic femur length 5.46; mesothoracic tibia length 4.59; mesothoracic tarsus length 3.85; metathoracic femur length 6.48; metathoracic tibia length 7.26; metathoracic tarsus length 5.30; anteroventral femoral spine count R12/L12; posteroventral femoral spine count R4/L4; anteroventral tibial spine count R11/L10; posteroventral tibial spine count R7/L8.

A DK cyclopean ear present on the ventral surface of the metathorax (see Yager and Svenson 2008 for description of the DK form).

**Figure 4.**
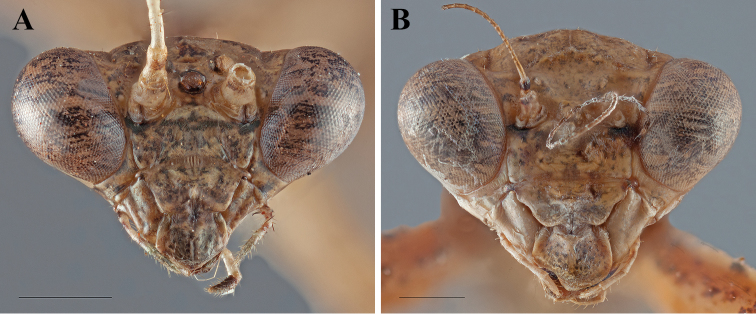
*Dystacta tigrifrutex* sp. n., head from the anterior perspective (scale bar = 1 mm): **A** holotype male **B** allotype female.

*Head* (Fig. 4A): Transverse, eyes slightly exophthalmic; the vertex rounded, the parietal sutures present; juxta-ocular protuberances absent. Frontal suture faint with a medial carina forming a continuous arc. Ocelli large, protruding on small cuticular mounds, but the region between all three slightly raised, with a triangular shape; the lateral ocelli oriented outward; the region around the raised ocelli and below the frontal suture depressed. Clypeus transverse, the upper margin convex, the lateral margins tapering; surface with moderate sculpting; the lower margin of the clypeus slightly concave. Labrum rounded. Antennae with a mostly pale pedicel and scape, but both with small black marks; the flagellomeres mostly pale in the basal half, transitioning to black on the distal end of the antennae, the setae dark colored, with 4-5 setae on each flagellomere. Anterior surface of head mostly pale or ochre with tiny dark speckling across the surface; frons ochre with dark splotches of black, a transverse black band just below the antennae extends laterally across the anterior surface of the eyes; the clypeus ochre with dark splotches of black; labrum ochre with black splotches, with ochre mandibles; vertex ochre with black splotches; raised area around ocelli black. Maxillary palpi ochre with black spots with the terminal segment black on one side.

**Figure 5.**
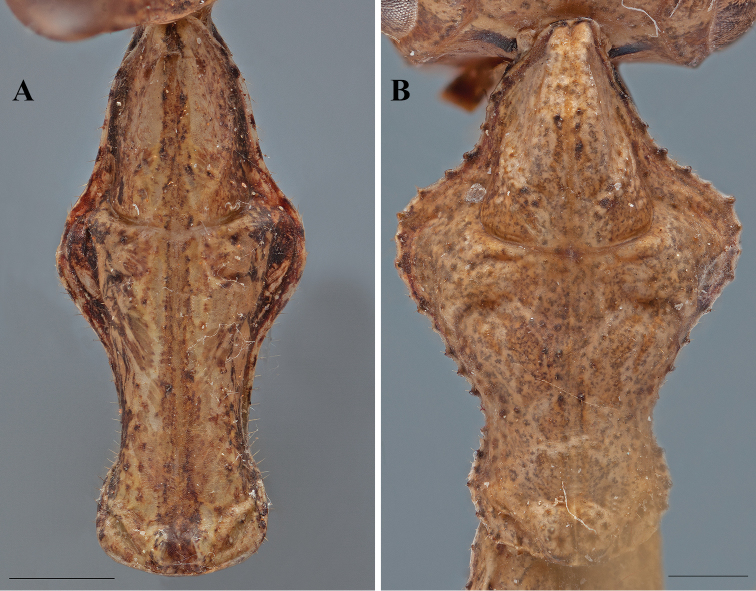
*Dystacta tigrifrutex* sp. n., pronotum from the dorsal perspective (scale bar = 1 mm): **A** holotype male **B** allotype female.

*Pronotum* (Figs 2B, 5A): Longer than wide, with an expanded supra-coxal bulge; dorsal surface smooth. Prozone with two angled carina oriented anterolateral from the supra-coxal sulcus; medial region of prozone peaked, sloping steeply to the anterior margins; medium length with margins gradually tapering anteriorly to a rounded anterior margin; the margins smooth, but with setae present. Metazone with two small depressions posterior to the supra-coxal sulcus; two large and prominent tubercles positioned on each side of the medial line just anterior to the posterior margin, a raised carina oriented anterolaterally, extending to the lateral margins of the metazone. Metazone with concave lateral margins tapering posteriorly until two thirds from the supra-coxal bulge, then widening to the posterior margin; margins smooth, but with setae present; the dorsal surface of the metazone not depressed. Ochre with faint black spots and more opaque black lines laterally. Prosternum ochre with dark brown speckling that becomes more dense posteriorly.

*Forelegs*: Femur shape normal with a straight dorsal margin; spines robust, pale proximally and black distally; femoral groove to accommodate the tibial spur in the proximal half; the posterior surface smooth; 4 discoidal spines. Posterior surface of femur ochre with black stippling; anterior surface ochre with black speckling that is more concentrated along the outer margins; ventral surface with a black band between the second most proximal and third most proximal posteroventral spines as well as a black band extending half way from the most distal posteroventral spine to the genicular spine; setae dispersed across a pale ventral surface. The discoidal spines robust, the third from the base very large and robust, twice the length of the second and fourth. Anteroventral femoral spines 1, 3, 5, 7, 9–11 short, but of similar length to each other; anteroventral femoral spines 2, 4, 6, 8 long, but of similar length to each other; posteroventral femoral spines all of the same length; the posterior and anterior genicular spines small, but robust. Tibia with numerous setae along the dorsal margin and on the posterior, anterior and ventral surfaces; anterior and posterior surfaces ochre with black stippling; posterior external surface ochre with groups of dense black stippling, occasionally with a black stippled line beginning at the base of the tibio-femoral joint to about a quarter of the way to the distal end to the tibia. Posteroventral tibial spines 1-6 of similar length, the distal spine 7 larger than the others; anteroventral tibial spines gradually increase in length from the most proximal to the most distal spine. Forecoxae mostly smooth with setae interspersed throughout, a few tubercles present along the margins, but none are robust as seen in females.

*Meso- and Metathoracic Legs*: Femora with pronounced ventral (posterior) carina; dorsal (anterior) carina faint. The femora, tibiae and tarsi with dense setae. Femora mostly pale with a number of black bands near the distal terminus on the posterior surface, the most pronounced running just dorsal to the ventral carina; anterior surface with a number of black bands along the ventral carina. Tibia pale with a number of faint black marks, some appearing as bands. Tarsi short; the first three tarsal segments are light proximally and have a dark spot on the distal end, the remaining segments black; mesotarsi and metatarsi with the first segment shorter than the remaining segments combined.

*Wings*: Elongate, extending well beyond the terminus of the abdomen. Forewing slender, the costal region narrow and opaque brown; dense setae along the anterior margin; the entire surface tightly ciliated; the discoidal region smoky grey with few dark splotches, three or four in the distal half; the veins more pigmented than surrounding cell colors, but small black sections disperse across all veins and occasionally along the major wing veins, increasing in frequency moving toward the distal terminus. Hindwings with setae along anterior margin, the surface tightly ciliated; matching coloration of forewing, but the costal region translucent grey in the proximal half, fading to opaque brown in the distal half; black marks along the distal terminus of the discoidal region; the terminus of the discoidal region projecting well beyond the margin of the anal region, the wing appearing highly elongate.

*Abdomen*: Smooth, tubular with brown and black coloration. A black medial line on the ventral surface that is contiguous across sternites; the posterior half of sternites fading from brown to black; the surface with numerous setae. Tergites rounded at the postero-lateral margins. Supra-anal plate and cerci not known since the posterior half was detached and carried away by opportunistic ants when drying.

*Genital Complex*: Due to specimen damage, the genitalia are absent.

**Female.** **Allotype** (Figs 1B, 3C–D). Female was preserved in ethanol and later pinned, causing some deformation in the abdomen. Body length 19.82; pronotum length 5.89; prozone length 2.56; pronotum width 3.83; pronotum narrow width 1.83; head width 4.83; head vertex to clypeus 3.72; frons width 1.87; frons height 1.10; prothoracic femur length 6.61; mesothoracic femur length 5.46; mesothoracic tibia length 4.82; mesothoracic tarsus length 3.91; metathoracic femur length 6.18; metathoracic tibia length 7.10; metathoracic tarsus length 4.73; anteroventral femoral spine count R12/L11; posteroventral femoral spine count R4/L4; anteroventral tibial spine count R11/L11; posteroventral tibial spine count R9/L9.

A DO cyclopean ear present on the ventral surface of the metathorax (see Yager and Svenson 2008 for description of the DO form).

*Head* (Fig. 4B): Slightly transverse, eyes not particularly exophthalmic; vertex highly convex, the parietal sutures present; juxta-ocular protuberances absent. Frontal suture faint, but a forming dorsally oriented curved. Ocelli absent or vestigial, but lenses not visible; a carina connecting former ocellar locations connecting all three and forming a U; region within the U and below the frontal suture depressed. Frons transverse with an angled carina below the antennae, meeting medially with a raised point. Clypeus transverse, the upper margin convex, the lateral margins tapering to a rounded distal terminus; surface with a medial, transverse carina. Labrum rounded with a darkened distal terminus. Antennae with a mostly pale pedicle that has a black band distally; scape black, flagellomeres pale basally and fading to black within 10 antennomeres; setae sparse basally, becoming more dense distally. Anterior surface of head mostly pale or ochre with tiny dark speckling across the surface; frons with a transverse black band just below the antennae; labial palpi ochre, maxillary palpi ochre then with a black spot on both sides of the terminal segment.

*Pronotum* (Fig. 5B): Longer than wide, with an expanded supra-coxal bulge; dorsal surface with uneven tiny depressions and scattered black tubercles. Prozone with two angled carina oriented anterolateral from the supra-coxal sulcus; medial region of prozone peaked, sloping steeply to the lateral margins. Metazone with uneven sculpting across the surface, two depressions posterior to the supra-coxal sulcus; two large and prominent tubercles positioned on each side of the medial line just anterior to the posterior margin. Margins of the prozone convex, tapering sharply to a narrowed, rounded anterior margin; margins of metazone strongly convex, tapering to a narrow constriction medially before widening to the posterior margin. Small, blunt tubercles present on the margins of the prozone; a few large tubercles present on the margins of the metazone, diagonally pointed setae protruding from the posterior side of the lateral half of the tubercle. The supra-coxal sulcus strongly defined and sweeping anteriorly prior to the expanded lateral margins at the supra-coxal bulge. Metazone provides the lateral expansion at the supra-coxal bulge; the dorsal surface flat, but bulging over the lateral margins in the anterior half. Prosternum ochre with dark brown speckling that becomes more dense posteriorly.

*Forelegs*: The femur squat, almost forming a triangle; spines robust, pale basally and black distally; femoral groove to accommodate the tibial spur just proximal to the middle. The posterior surface of the femur with a marginal carina; dorsal margin narrowing, almost lamellar along the slightly convex margin; dorsal margin with small tubercles that give rise to small hairs; the posterior surface with numerous small tubercles; 4 discoidal spines. The ventral surface of the femur pale with numerous tubercles medially distal to the discoidal spines, each giving rise to a long hair, tubercles continue just proximal to the discoidal spines until the junction with the trochanter; the discoidal spines robust, the third from the base very large and robust. The anterior surface of the femur mostly pale, but with black markings along the ventral margin anterior and posterior to the femoral groove, the groove itself pale; black speckling across the surface. Anteroventral femoral spines (proximal to distal) 2, 4, 6, 8, 12 long, but of similar length to each other, spines 1, 3, 5, 7, 9-11 short, but of similar length to each other; posteroventral femoral spines all of similar length; the posterior and anterior genicular spines small, but robust. Tibia robust with rare, fine setae on the surface and near the lateral margins of the ventral surface; posterior surface ochre with black stippling, ventral surface ochre and lustrous; posteroventral and anteroventral tibial spines gradually becoming longer from the proximal to the distal end. Coxae with tubercles and setae across the surface, the dorsal margin with setae and a few strong tubercles; the anterior surface mostly ochre with small black spots, a large black marking near the distal margin, the distal lobes ochre.

*Meso- and Metathoracic Legs*: Femora with ventral (posterior) carina well developed; dorsal (anterior) carina absent; surface with numerous small, fine setae; darkly speckled with black markings, sparse proximally, becoming more dense distally, but with a pale band in the distal half. Coxae with numerous black markings speckling the surface. Tibia round, covered with setae; mostly black with two pale bands, one in the proximal half and the other in the distal half. Tarsi short with ample setae; mostly black with the first three tarsal segments are light proximally and darken toward the distal end, the remaining segments black; mesotarsi and metatarsi with the first segment shorter than the remaining segments combined.

*Wings*: Apterous with no visible vestiges.

**Figure 6.**
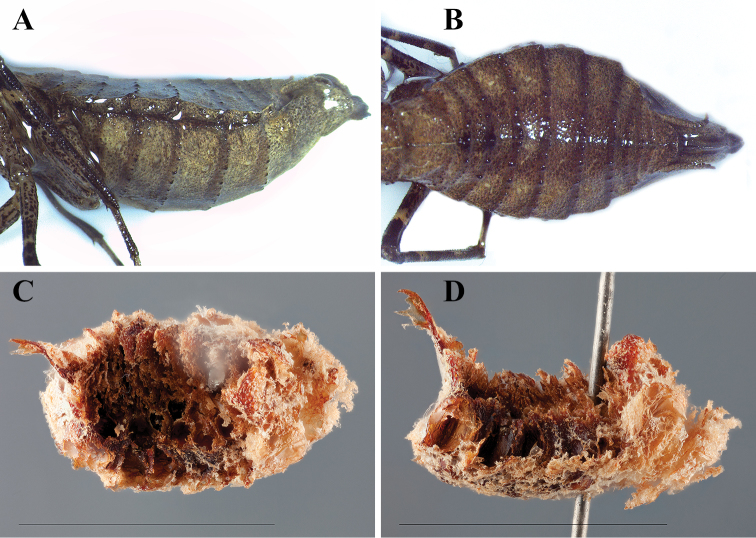
*Dystacta tigrifrutex* sp. n. Allotype female abdomen preserved in ethanol: **A** lateral **B** dorsal. Ootheca (scale bar = 1 cm): **C** dorsal **D** lateral.

*Abdomen* (Fig. 6A–B): Very broad, elliptical, the widest being the middle. Tergites and sternites with small, black tubercles along the posterior margins; fine setae disperse across dorsal and ventral surface; each tergite with a medial keel, more pronounced anteriorly. Described from the ethanol preserved specimen, plates 2,3,4, and 5 with small carinae near the lateral margin forming a 45 degree angle with the distal margins of their respective segments, the carinae highest at the margin and descending medially at approximately a 45 degree angle. Tergites rounded at the postero-lateral margins. Supra-anal plate transverse, evenly rounded terminus. The ovipositor enlarged and broad, projecting far beyond the distal margin of the supra-anal plate and the cerci. Cerci round, tapering to a point.

**Nymphs.** *First Instar*. Length 3.89; pronotum length 1.21; prozone length 0.52; pronotum width 0.73; pronotum narrow width 0.43; head width 1.39; prothoracic femur length 1.61; mesothoracic femur length 1.38; mesothoracic tibia length 1.28; mesothoracic tarsus length 1.23; metathoracic femur length 1.89; metathoracic tibia length 1.79; metathoracic tarsus length 1.29; anteroventral femoral spine count R12/L12; posteroventral femoral spine count R4/L4; anteroventral tibial spine count R10/L10; posteroventral tibial spine count R8/L8.

*Head*: Transverse, eyes exophthalmic; vertex highly convex, parietal sutures present; juxta-ocular protuberances absent. Frontal suture forming a dorsally oriented curve. Ocelli not fully developed; the anterior ocellus raised; the location of the lateral ocelli marked by yellow bumps, connected by a U-shaped carina; the region within the U and below the frontal suture slightly depressed. Frons transverse with an angled carina below the antennae, meeting medially with a slightly raised point. Clypeus transverse, the upper margin convex, the lateral margins tapering; surface with a medial, transverse carina. Labrum rounded with a darkened distal terminus. Antennae with a faded black pedicel; scape pale with black splotching; flagellomeres pale basally and fading to black within four antennomeres; setae very sparse basally, becoming slightly more dense distally. Anterior surface of head dark brown with light brown spots; a pale ring formed around the circumference of the antennal insertion sites and a pale arc formed between the eyes posterior to the ocelli. Labial and maxillary palpi segments black basally with a distal light brown terminus.

*Pronotum*: Longer than wide, with an expanded supra-coxal bulge; dorsal surface smooth. Prozone with a strongly peaked medial region, sloping steeply to the lateral margins. Metazone smooth with a continuation of the peaked region of the prozone sloping steeply to the lateral margins; two small bumps are found on either side of the midline at the distal end of the metazone.

*Forelegs*: The femur squat, nearly triangular; spines robust with a blackish-brown coloration; femoral groove to accommodate the tibial spur proximal to the middle. The posterior surface of the femur with a marginal carina; dorsal margin narrowing, almost lamellar along the slightly convex margin; dorsal margin with small tubercles that give rise to small hairs. The ventral surface blackish-brown with occasional tubercles giving rise to small hairs; the discoidal spines robust, the third from the base very large and robust. Anterior surface of femora blackish-brown; the groove blackish-brown as well. Anteroventral femoral spines (proximal to distal) 2, 4, 6, 8, 12 are long and of similar length to each other while spines 1, 3, 5, 7, 9-11 are short and of similar length to each other; posteroventral femoral spines all of similar length; the posterior and anterior genicular spines small, but robust. Tibia robust with several setae on the anterior surface and near the lateral margins of the ventral surface; posterior surface ochre; posteroventral and anteroventral tibial spines gradually becoming longer from the proximal to the distal end. Coxae smooth; the surface ochre with blackish-brown spots and a blackish-brown band at the distal end; the distal lobes ochre.

*Meso- and Metathoracic Legs*: Femora with ventral (posterior) carina; dorsal (anterior) carina absent; surface with numerous small, fine setae; ochre with faint, small brown spots and three wide, faint brown bands. Coxae ochre. Tibia round, covered with setae of varying size; dark brown with four sand colored bands. Tarsi short and covered with setae; black and blackish-brown in color; mesotarsi and metatarsi with the first segment shorter than the remaining segments combined.

*Abdomen*: Broad, elliptical, widest in the middle. Tergites with a medial keel, more pronounced anteriorly. Setae occurring on the lateral margins projecting laterally. Tergites rounded at the postero-lateral margins. Supra-anal plate transverse. Cerci round and short, tapering to a point.

#### Ootheca

(Fig. 6C–D). *Measurements*. Length 11.12; width 6.40; height 5.24; perimeter (dorsal perspective) 33.87; perimeter (from lateral perspective) 43.23. From the dorsal perspective, the ootheca appears elliptical, with a distinct dorsal point on the posterior end where the egg-case laying terminated. The ootheca is convex ventrally and concave dorsally from the lateral perspective. The anterior end is larger than the remainder of the ootheca, which is consistent in girth with the exception of the terminal dorsal point. Large egg chambers can be seen in rows perpendicular to the top and bottom of the egg-case. The emergence area takes up the majority of the dorsal surface. There is very little air space, the egg chambers extending nearly to the perimeter of the oothecae. The interior of the ootheca is a dark to light reddish brown with a foam textured light brown cast covering the external surface. The method of attachment for this species appears to be smooth vertical surfaces, with no apparent ventral circlet at the point of attachment. This hypothesis is based off the observation of a single specimen that laid its ootheca on the side wall of a mesh cube container in which it was temporarily held (the flexible surface may have influenced the convexity of the ventral surface). The cube contained several twigs at various angles as well as leaves.

#### Etymology.

The word *tigrifrutex* is derived from the latin word *tigris* (meaning tiger) and the latin word *frutex* (meaning bush). This name was crafted to reflect the behavior of the female, whose morphology suggests that she is adapted for hunting prey close to the ground and in the undergrowth.

## Supplementary Material

XML Treatment for Dystacta

XML Treatment for Dystacta alticeps

XML Treatment for Dystacta tigrifrutex
